# Microbial Biotechnology is 15! An adventure in journal parenting

**DOI:** 10.1111/1751-7915.13984

**Published:** 2021-11-30

**Authors:** Kenneth Timmis, Joan Timmis

**Affiliations:** ^1^ Institute of Microbiology Technical University of Braunschweig Braunschweig Germany

## Abstract

This year, 2022, *Microbial Biotechnology* (*MBT*) celebrates its 15th birthday. In journal terms, 15 is adulthood. It has been a privilege to develop the idea, launch the journal, nurture it in its infancy and stimulate and support it during its adolescence and early adulthood. The success of *MBT* – its growth over the last 4 years averaging > 30%, and the highest impact factor in the field, making it the leading research journal in applied microbiology/microbial biotechnology and the most attractive to publish in – gives us enormous pleasure and satisfaction, and not a little pride.

The idea to create *MBT* originated in the increasing number of submissions to *Environmental Microbiology* (*EMI*) reporting research having a strong applied content and only a tenuous link to the environment. *MBT* was therefore developed as a sister journal to *EMI* for publication of research in applied microbiology. Preparations began in 2007 and the first issue was published in 2008 (https://sfamjournals.onlinelibrary.wiley.com/toc/17517915/2008/1/1). We had a star team of *Editors*: Juan Luis Ramos, Marty Rosenberg, Willem de Vos and Willy Verstraete, with Kenneth Timmis as EiC. From the beginning, we published the features *Genomics Update*, with Roland Siezen as Editor (see, e.g. https://sfamjournals.onlinelibrary.wiley.com/doi/epdf/10.1111/j.1751‐7915.2008.00030.x), and *Web Alert*, with Larry Wackett as Editor (see, e.g. https://sfamjournals.onlinelibrary.wiley.com/doi/epdf/10.1111/j.1751‐7915.2007.00019.x). And we were able to appoint an equally stellar *Editorial Board* of experts to review manuscript submissions. Joan Timmis served as Journal *Administrator*, checking submissions, dealing with queries from all and sundry, steering, solving problems, advising, putting out fires and generally making lives of *Editors*, *Reviewers* and *Authors* more efficient and enjoyable. Over the 15 years since the launch, Willem, Willy, Marty and Roland cycled off and were replaced by Siegfried Vlaeminck (also cycled off), Auxi Prieto, Harald Brüssow, Hui Wang, Pablo Nikel, Tom Clavel and Antoine Danchin (*Genomics Update*; see, e.g. this issue). In addition, Wei Huang has ably contributed to the editing of synthetic microbiology submissions.

The aim of *MBT* was not simply to passively publish work on applied microbiology but rather to actively stimulate and support the field, and steer it towards new and exciting topics, especially those involving original and/or synergistic multidisciplinary approaches. To accomplish this, *MBT* features not only primary research articles on any aspect of applied microbiology but also *Special Issues* on emerging topics. These are complemented and supported by *MiniReviews* and *Editorials* solicited from leading players. Exciting articles that we publish are frequently flagged up in *Highlights* that are now written or commissioned by the *Highlights Editor*, Carmen Michan. Every two years, we publish our *Crystal Ball* feature, in which leaders in the field speculate on the nature of the most important developments driving future advances (see, e.g. https://sfamjournals.onlinelibrary.wiley.com/toc/17517915/2019/12/1). This feature is not only very stimulating and a significant source of discussion, but also a lot of fun for both authors and readers. Juan Luis has made outstanding contributions to MBT as *Special Issues* and *Minireviews Editor*, in addition to his responsibility as a regular *Editor*.

In 2017, *MBT* published a *Special Issue* on *The Contribution of* *Microbial Biotechnology* *to Sustainable Development Goals* (http://onlinelibrary.wiley.com/doi/10.1111/mbt2.2017.10.issue‐5/issuetoc; https://sfamjournals.onlinelibrary.wiley.com/doi/epdf/10.1111/1751‐7915.12818), which assessed the enormous potential of diverse fields of applied microbiology to contribute to attainment of most of the SDGs (i.e. SDG2, 3, 6, 7, 8, 11, 12, 13, 14, 15). This *Special Issue* generated considerable interest, not only because it discussed, and revealed for the first time for some, how microbiology is currently impacting and will impact sustainability, thereby describing a framework for microbiology‐centric sustainability research and action, but also encouraged and enabled young microbiologists to see their work in a wider context and its relevance to sustainability. This was a pioneering *Special Issue* that triggered a number of journals and microbiology societies to subsequently paint sustainability and the SDGs on their banners.

Another pioneering *Special Issue* was on the topic of Microbial Biotechnology *in China* (https://sfamjournals.onlinelibrary.wiley.com/toc/17517915/2021/14/2), both to recognise and reveal the enormous and rapidly growing importance of Chinese research in the field, and its priorities (https://sfamjournals.onlinelibrary.wiley.com/doi/epdf/10.1111/1751‐7915.13777). The *Special Issue* on *Synthetic Biology beyond Borders,* which constitutes the last issue of 2021 is also outstanding (https://sfamjournals.onlinelibrary.wiley.com/toc/17517915/current)!

More recently, *MBT* has together with *EMI* been publishing a new feature entitled *Lilliput* contributed by Harald Brüssow. Although his initial intention was to summarise latest findings in exciting emerging areas, in *MBT* they have so far focussed entirely on the COVID‐19 pandemic. As a trained virologist, Harald is well equipped to digest latest developments and present them to the *MBT* readership. The *Lilliputs* thus far published represent an outstanding and comprehensive series of summaries of latest findings, ranging from the origins of the infection, its transmission, clinical disease, vaccine and therapy developments, and their corresponding clinical trials. The latest *Lilliputs*, assessing the relevance of the 1889 *Russian flu* to the trajectory of the COVID‐19 pandemic (https://sfamjournals.onlinelibrary.wiley.com/doi/epdf/10.1111/1751‐7915.13916), also in the context of the possibility that the *Russian flu* pandemic may have been caused by a coronavirus (https://sfamjournals.onlinelibrary.wiley.com/doi/epdf/10.1111/1751‐7915.13889), are examples of these outstanding feature articles, that are unique to *MBT*.

To support and encourage young investigators to the field to explore career opportunities in important and emerging fields, *MBT* published a series of *Editorials* in 2017 and 2018 on the topic of *The microbiome as a source of new enterprises and job creation* (see, e.g. https://sfamjournals.onlinelibrary.wiley.com/doi/epdf/10.1111/1751‐7915.12877), and in 2019 a series of editorials on the topic of *Synthetic microbiology as a source of new enterprises and job creation* (see, e.g. https://sfamjournals.onlinelibrary.wiley.com/doipdf/10.1111/1751‐7915.13372).

Being at the helm of *MBT* for 15 years and having the fun of working with the leaders and soon‐to‐be leaders in the field, whether as *Editors*, *Reviewers* or *Authors*, reading about their exciting discoveries, and following their careers and the contributions they make and the recognition they receive, has provided us with enormous pleasure and satisfaction. But, 15 years is long enough and it is time for new blood and ideas so, as *MBT* enters its 15th year, we will cycle off. To some extent, we feel like Mum and Dad of both the Journal and the brilliant young things who fill it with exciting discoveries. And like mums and dads should, we rejoice, knowing that we are handing on a success story to the next generation.

And so we are absolutely delighted that Juan Luis accepted to become the new EiC! *MBT* is in the best possible hands: Juan Luis has some 30 years of continuous experience in editing top journals: first *J. Bacteriol*., then *EMI* and, from its launch, *MBT*. But, though impressive, it is not experience that is the most important quality he brings: he is a visionary, brilliant strategist, creative, has an enormous capacity for work, and is collegial, inclusive and persuasive, all of which enable him to get his visions realised. He has already placed his stamp on the future *MBT* with an excellent strategic plan and recruitment of two brilliant new, young and dynamic Editors—Sepideh Pakpour and Davinia Salvachua: welcome Davinia and Sepideh! Thank you Juan Luis for your wonderful partnership over the years. *MBT* will grow mightily under your leadership.

## Dedication

This Editorial is dedicated to the wonderful Editors of MBT, the members of its Editorial Board, its *ad hoc* reviewers, its authors, and some special folk we call *Friends of MBT*, who generously provide council and highly original submissions, like Victor de Lorenzo, Willy Verstraete, Willem de Vos, and others. Thank you all!

